# Measuring antigen expression of cancer cell lines and circulating tumour cells

**DOI:** 10.1038/s41598-023-33179-y

**Published:** 2023-04-13

**Authors:** Anouk Mentink, Khrystany T. Isebia, Jaco Kraan, Leon W. M. M. Terstappen, Michiel Stevens

**Affiliations:** 1 Medical Cell Biophysics Group, Techmed Center, Faculty of Science and Technology, University of Twente, PO Box 217, 7500 AE Enschede, The Netherlands; 2 Department of Medical Oncology, Erasmus MC Cancer Institute, Erasmus University Medical Center, Rotterdam, The Netherlands

**Keywords:** Cancer, Metastasis, Tumour biomarkers, Tumour heterogeneity

## Abstract

When evaluating EpCAM-based enrichment technologies for circulating tumour cells (CTCs), the cell lines used should closely resemble real CTCs, meaning the EpCAM expression of CTCs needs to be known, but also the EpCAM expression of cell lines at different institutions and times is important. As the number of CTCs in the blood is low, we enriched CTCs through the depletion of leukocytes from diagnostic leukapheresis products of 13 prostate cancer patients and measured EpCAM expression using quantitative flow cytometry. Antigen expression was compared between multiple institutions by measuring cultures from each institution. Capture efficiency was also measured for one of the used cell lines. Results show CTCs derived from castration-sensitive prostate cancer patients have varying but relatively low EpCAM expression, with median expression per patient ranging from 35 to 89,534 (mean 24,993) molecules per cell. A large variation in the antigen expression of identical cell lines cultured at different institutions was found, resulting in recoveries when using the CellSearch system ranging from 12 up to 83% for the same cell line. We conclude that large differences in capture efficiency can occur while using the same cell line. To closely resemble real CTCs from castration-sensitive prostate cancer patients, a cell line with a relatively low EpCAM expression should be used, and its expression should be monitored frequently.

## Introduction

Cell lines are often used to compare different methods, or optimize protocols for the enrichment and isolation of circulating tumour cells (CTCs). The cell lines used are stated in some articles or reviews where several enrichment technologies are compared^1–4^. This makes for a seemingly more fair comparison, as characteristics such as size, deformability and antigen expression can differ widely between different cell lines. Especially the latter characteristic is of high importance when evaluating antigen-based enrichment technologies.

When using a surface antigen for selective rare cell capture, a large number of interactions between the coated surfaces and the cells need to be generated, while also allowing for the retainment of the captured cells. The most used method for this is immunomagnetic capture, where (super-para-)magnetic beads are coated with an antibody^5^. For CTC capture, in most cases, the antigen of relevance is the epithelial cell adhesion molecule (EpCAM), an antigen that is overexpressed in most cancers of epithelial origin in both tumour tissue and CTCs^6^. An example of immuno-magnetic enrichment is the CellSearch system, which is the most used method for CTC enrichment^7^.

The efficiency of such an enrichment is largely dependent on the number of antigens present on the cell surface. Therefore cell lines with comparable expression characteristics should be used when evaluating these technologies, thereby more closely mimicking real CTCs. Here not only characteristics used for capture, but also those used for the identification of CTCs should be considered. The cytokeratin (CK) expression level of the often used LNCaP cell line has for instance been shown to be much higher than that of patient CTCs^8^, making their identification unrealistically easy.

Using a cell line with a realistic EpCAM expression is even more relevant given the more recent insight into the existence of a subpopulation of EpCAM-low or even EpCAM-negative CTCs^9–13^. Several studies have already attempted to increase the sensitivity of their antigen based capture methods, either by the use of additional antigens^14,15^, or by increasing the magnetic force generated^16–18^, while many have also focussed on completely EpCAM independent capture methods^19^. These methods are all designed to also capture low EpCAM expressing cells; even the CellSearch system uses a specialized patented approach to bind sufficient magnetic particles to CTCs with low EpCAM expression^20^.

As any increase in sensitivity will generally be made at the cost of specificity, it would be beneficial to know the needed sensitivity of such a system. For this the antigen expression level of CTCs in patient samples needs to be known. Although there are several studies in which the expression of EpCAM on CTCs is evaluated^21–23^, to the best of our knowledge only Rao et al. have attempted to quantitatively measure the EpCAM expression of CTCs^24^, showing large differences between the measured EpCAM expression and several commonly used cell lines. In this work, they however set a minimal EpCAM expression threshold for the identification of CTCs, thereby excluding any EpCAM low or negative CTCs. This means that quantification of the EpCAM expression on the total CTC population is needed to be able to effectively select the most representative cell lines, allowing a realistic determination of the sensitivity of any EpCAM-based capture assay.

For this purpose, not only the EpCAM expression in CTCs, but also that of several cell lines will need to be known. As cell characteristics in-vitro are known to change over time^25^, not only the current level of antigen expression is of importance, also the variation is of note. This variation could likely be minimized by wide adherence to the recommended culture conditions of the ATCC. In practice however, institution dependent culture conditions often are applied, potentially leading to large differences between institutions. To test this, we measured the expression of several cancer related antigens on different cell lines when cultured using our own standard culture conditions, as well as on cells that have been cultured at different institutions using their standard culture conditions.

As CTCs in especially non-metastatic, but also early metastatic cancer are very rare^26^, measurement of their characteristics is challenging. A way of attaining a larger number of CTCs is through the use of leukapheresis^27^, which allows the interrogation of larger blood volumes. Therefore, with the aim to quantitatively measure their EpCAM expression level, we have identified prostate cancer CTCs in leukapheresis-derived patient samples using non-EpCAM markers. Furthermore, we have compared their EpCAM expression to that of several often used cancer cell lines.

## Results

To quantify the expression of different antigens, cells were stained using monoclonal antibodies coupled to phycoerythrin (PE), after which the number of PE molecules per cell was determined using quantitative flow cytometry. Although the number of PE molecules will not be exactly equal to the number of antigens, in this work we will treat the number of PE molecules per cell as being representative of the number of antigens expressed.

### Sample preparation and staining

**Figure 1:**
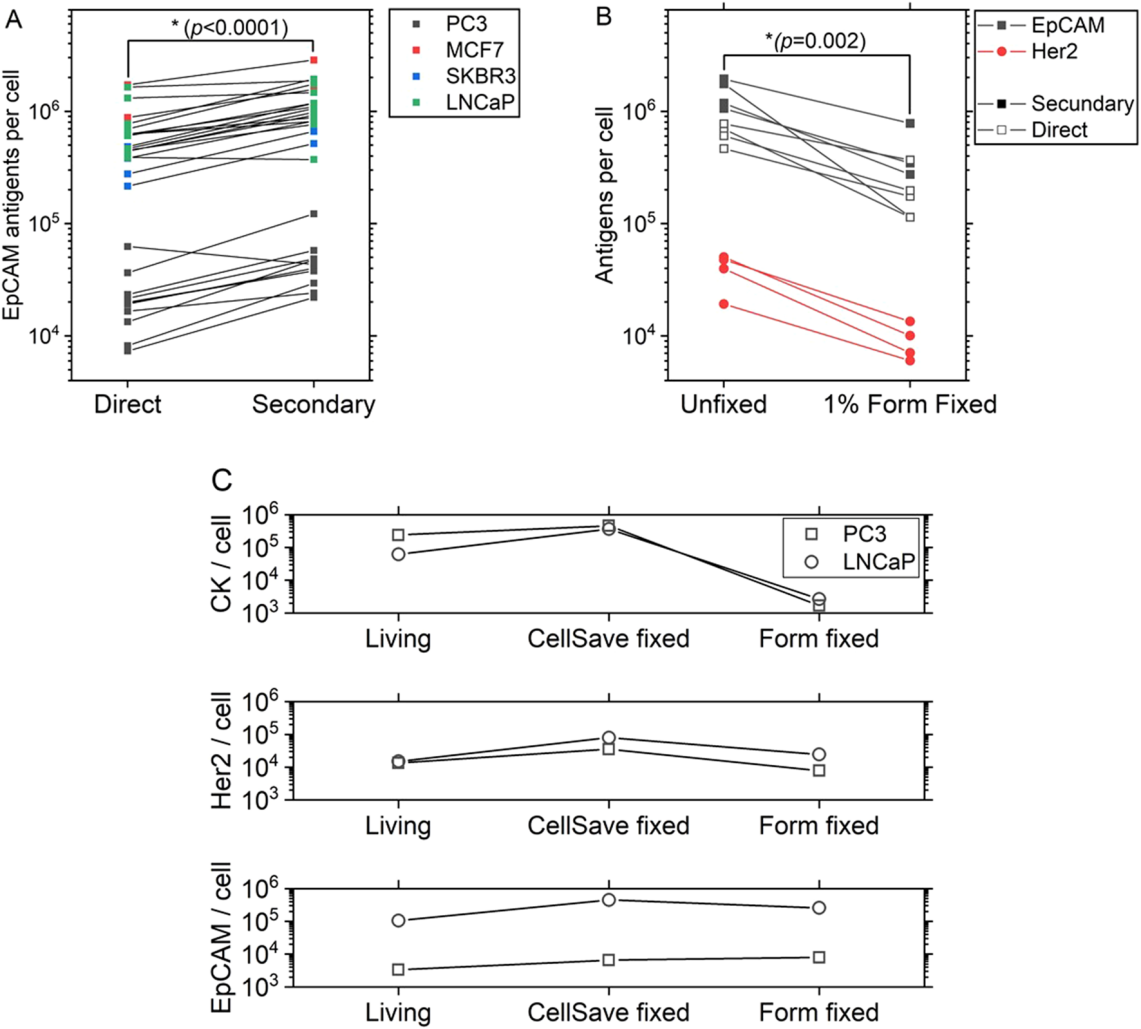
(**A**) EpCAM expression of unfixed PC3 (N = 11), MCF7 (N = 4), SKBR3 (N = 4) and LNCaP (N = 11) measured either using a directly conjugated antibody or by secondary antibody staining. (**B**) EpCAM and Her2 expression of LNCaP cells measured using unfixed or 1% formaldehyde fixed cells (N = 3). (**C**) CK, Her2 and EpCAM expression measured of unfixed, CellSave fixed and 1% formaldehyde fixed LNCaP and PC3 cells after 24 h (N = 1).

Antigen expression can be measured either directly, using an antibody with a conjugated fluorophore, or via an unconjugated antibody that is then tagged using a secondary antibody which is conjugated to a fluorophore. Figure 1A shows that the measured expression in all five cell lines used is lower when the EpCAM antigen is labelled using a directly conjugated antibody compared to using a secondary antibody staining (Wilcoxon Signed Ranks Test, *p* < 0.0001). This is expected, as when using a polyclonal secondary antibody (e.g. anti-mouse-PE), more than one antibody can bind to the primary antibody resulting in a higher PE signal. This phenomenon concurs with the difference in the quantified expression, which differs up to a factor of three (mean 2.1, range 0.7–3.6). As this variation is seen in all cell lines, the relative expression differences between the cell lines remain similar.

The time between sample collection and antigen quantification in a patient sample is in our case approximately 24 h, meaning the antigen expression should be preserved over this period. In many cases, this is achieved through fixation^28^, either using a mild fixative such as CellSave (Menarini-Silicon Biosystems) or Cytochex (Streck), or a harsher fixative such as Transfix (Caltag Medsystems) or 1–4% formaldehyde (Merck). The effect a harsh fixation has on the measured expression of EpCAM and human epidermal growth factor receptor 2 (Her2) was determined in four independent experiments. In Fig. 1B it can be seen that a significant reduction (Wilcoxon Signed Ranks Test, *p* = 0.002) in the measured antigen expression is found upon cell fixation, with an average decrease of 72% (range 52–93%). We also tested the expression of CK, Her2 and EpCAM after 24 h using unfixed cells stored at 4 °C, CellSave fixed cells and 1% formaldehyde fixed cells. Results in Fig. 1C show a higher preserved expression of EpCAM and Her2 using CellSave compared to unfixed cells, while the formaldehyde fixation resulted in a large decrease for the intracellular cytokeratin, likely caused by the permeabilization agent not being able to sufficiently permeabilize the cells’ crosslinked membrane to allow passaging of the relatively large PE-fluorophore.

### Cell line antigen expression variability

**Figure 2:**
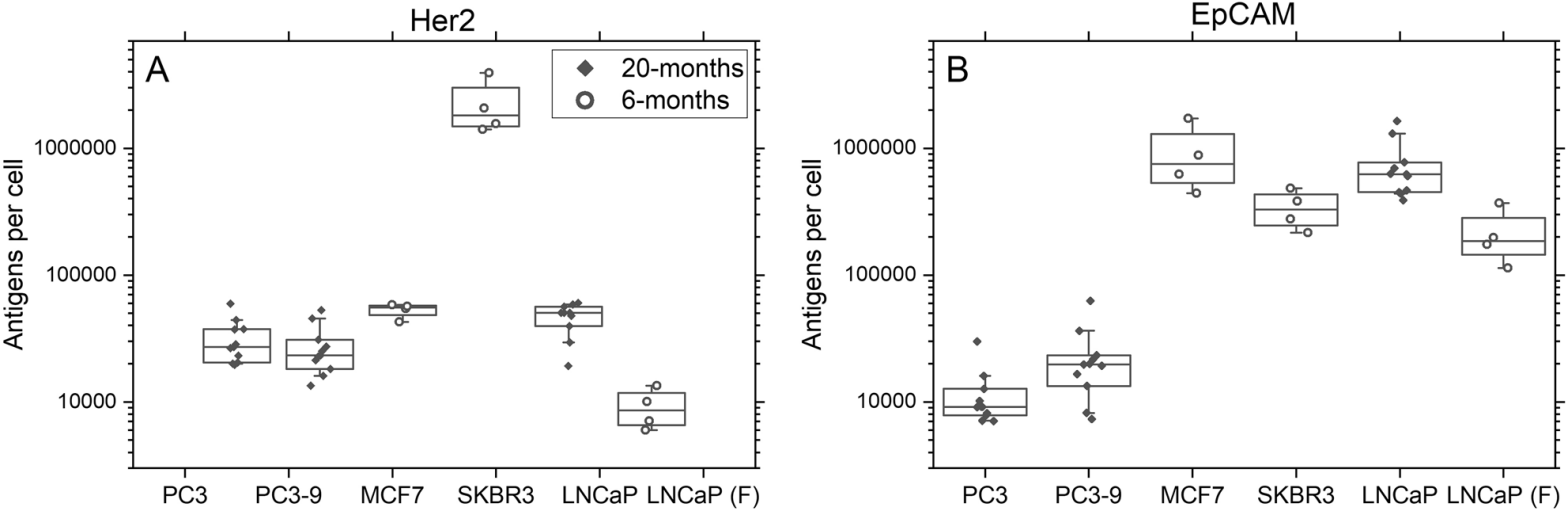
Expression of (**A**) Her2 and (**B**) EpCAM in unfixed PC3, PC3-9, MCF7, SKBR3, LNCaP as well as formaldehyde fixed LNCaP measured over a 6 or 20-month period. Boxes indicate 25–75 percentile with the median as a horizontal line, whiskers indicate 10–90 percentile.

We determined the expression of EpCAM and Her2 every two months on cells from the PC3, PC3-9 and LNCaP cell lines over a 20-month period (11 measurements), and cells from the MCF7 and SKBR3 cell lines over a 6-month period (four measurements), Fig. 2. Here, statistically significant differences (p < 0.02) in the expression of Her2 were found between all combinations of cell lines except from when comparing PC3 with LNCaP (p = 0.17), PC3 with PC3-9 (p = 1) or LNCaP with MCF7 (p = 1). The EpCAM expression showed a significant difference between both PC3 and PC3-9 compared to all other cell lines (p < 0.01). No significant difference was found between the EpCAM expression of PC3 and PC3-9 (p = 0.07), LNCaP and SKBR3 (p = 0.20), MCF7 and LNCaP (p = 1) or MCF7 and SKBR3 (p = 0.14), indicating that for these cell lines, the variation in the measured expression within the same cell line is in this dataset to large to significantly distinguish the difference in expression between these cell lines.

To asses if the variation seen within each cell line was caused by changes in the antigen expression or by experimental variation, we also measured Her2 and EpCAM expression on a fixed sample of LNCaP cells during the same 6-month period, see Fig. 2. The median Her2 expression of unfixed LNCaP cells was 46,523 molecules per cell but reduced by 80% to 9133 antigens in the fixed sample (*p* < 0.01), which can be attributed to the effect of fixation as seen in Fig. 1B. However, the coefficient of variation (CV) of Her2-expression for the fixed sample was only slightly higher than that of the unfixed sample (37% vs 27%). Similarly, the median EpCAM expression of unfixed LNCaP cells was 728,738 antigens per cell and decreased by 71% to 213,430 antigens per cell for the fixed LNCaP cells (*p* < 0.01). The CV of the EpCAM expression in the fixed sample (51%) was however similar to the CV of the unfixed sample (54%). The CV of the Her2 expression of all the cell lines ranged from 13 to 52% and for EpCAM from 30 to 75%.

Considering the similar variation observed in the fixed samples compared to the unfixed samples, the main cause of the measured variation in Her2 and EpCAM expression can be attributed to variations in the measurement and not to changes in the actual cell line. In Supplementary Fig. S1, the same datapoints are shown in time, where also a similar pattern of expression changes can be seen for multiple cell lines, which is in line with the changes being mostly a result of inter-experimental variation. It is however also clear from this data that the differences in expression between these cell lines are much greater than the measurement variability we found, indicating that albeit no exact quantifications can be obtained, this measurement can be used to determine the antigen expression range.

### Intra-experimental variation

From Fig. 2 it becomes clear there exists a relatively large variation in the measured expression between different experiments conducted at approximately two-month intervals. To determine to what extent this affected the comparison of expressions in a single experiment, we determined the intra-experimental variation by measuring the expression of EpCAM and Her2 on PC3-9 and LNCaP cells using triplicate samples within the same experiment, and the short-term inter-experimental variation using three replicates within the same week. For this, we used a batch of fixed cells to avoid variation in culture conditions. Results are shown in Supplementary Fig. S2 and show that for triplicate experiments performed on the same day, the intra-experimental variation of the highly expressed EpCAM antigen in LNCaP cells is relatively low with a CV of 5.6% but increases for antigens with lower expression up to 144% for the Her2 expression in PC3-9. Similarly, the inter-experimental variation is 20% for the EpCAM antigen in LNCaP cells and increases to 94% for Her2 antigens in PC3-9 cells. This confirms that it is possible to determine the antigen expression range of cells with low antigen expression, as well as multi-fold changes in medium- to high- expressing cell lines. However, the quantification of especially low antigen expressions should be viewed as a confirmation of the antigen expression range, not as a certain determination of the cells exact antigen expression level.

### Antigen expression per institution

Many institutions use the same cell lines, seemingly allowing for the direct comparison of for instance immunomagnetic enrichment technologies. Institutions however often have cell lines in culture for a prolonged time, and over time the antigen expression of these cells can vary, either as a result of different culture conditions or simply due to stochastic sampling at passage. To measure the differences that exist in the expression of several tumour-related antigens we compared their expression levels on four cell lines from at least three institutions as well as a vial purchased directly from the American Type Culture Collection (ATCC). For this, an aliquot of the cell lines was frozen in liquid nitrogen at each site and shipped to the University of Twente. Supplementary Table S1 shows culturing details for each institution and passage number of the cells at the time of measurement. In most cases, institutions use their own culture methods which may deviate from the recommendations by ATCC. At the University of Twente, all cultures of one of the cell lines were defrosted and allowed to grow for approximately 1 week. Then, in a single experiment, the antigen expressions of all cultures of that cell line were determined. In this way, for each cell line, the antigen expressions of all available cultures were measured in a single experiment.

**Figure 3:**
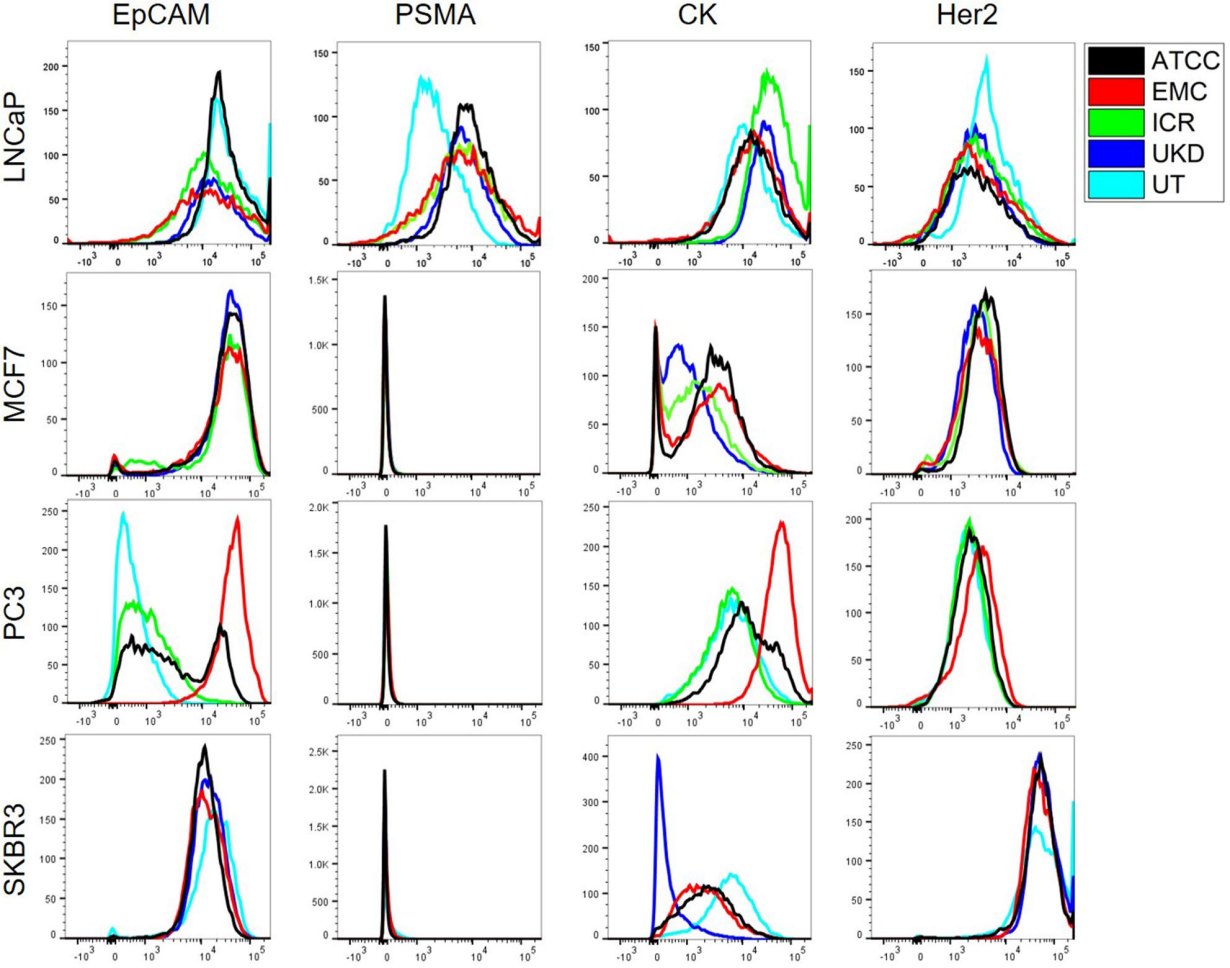
The expression of EpCAM, Her2, PSMA and Cytokeratin (CK) in LNCaP, MCF7, PC3 and SKBR3 from the ATCC, Erasmus MC (EMC), London Institute for Cancer Research (ICR), Universitätsklinikum Düsseldorf (UKD) and University of Twente (UT). Histograms of the PE signal are shown as measured using flow cytometry.

Figure 3 shows the histograms of the PE signal as measured using flow cytometry. In Supplementary Fig. S3 the corresponding mean number of antigens is depicted for each antigen and cell line. Several differences in the expression of these antigens between the cultures as kept at different institutions can be seen, such as the reduced expression of PSMA in the LNCaP cell line at the University of Twente or the large variation in the CK expression in all cell lines. In the CK expression of the SKBR3 and the EpCAM expression of the PC3 cell lines it appears that in these cell lines, the culture at the ATCC from which all others originate, already contains two subpopulations. Although the Erasmus and University of Twente use the same culture conditions for PC3 and they have similar passage numbers, the high EpCAM expressing subpopulation seems to have proliferated at the Erasmus, while the low expressing subpopulation has become dominant at the University of Twente.

### Effect on immunomagnetic capture

As EpCAM is the antigen that is mostly used to capture CTCs, a variation in the expression of this antigen is expected to lead to a difference in the perceived capture efficiency of CTC enrichment technologies. To test this, we spiked exact numbers of PC3 cells from each of the four institutions into blood samples from healthy donors. We then processed these samples using the CellSearch system and determined the recovery of PC3 cells for each institution.

**Figure 4:**
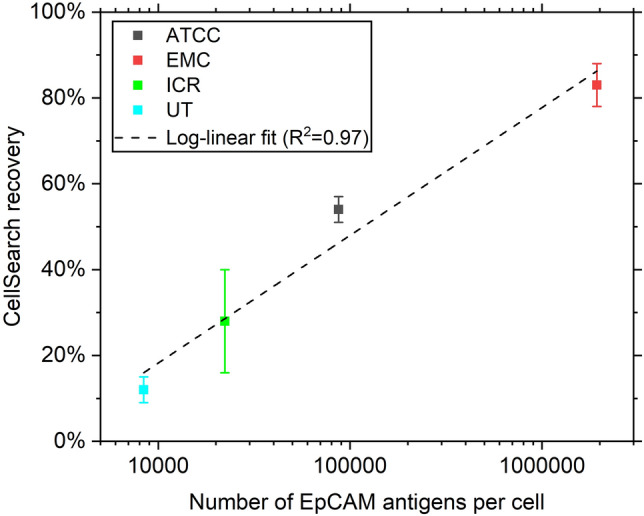
EpCAM expression of PC3 from the ATCC, Erasmus MC (EMC), London Institute for Cancer Research (ICR) and University of Twente (UT) together with the CellSearch recovery of each of the cell lines. N = 3. Error bar indicates mean ± SD.

In Fig. 4 the measured EpCAM expression as well as the CellSearch recovery of PC3 cells from each of the four origins is shown. The recovery ranges from 12% for PC3 cells cultured at the University of Twente up to 83% for PC3 cells cultured at the Erasmus MC. Linear regression after log-tranformation of the EpCAM expression resulted in a regression of CS_rec_ = − 101 + 30∙log (EpCAM_expr_) with a R^2^ of 0.97. To confirm the cells are indeed PC3 cells, Short Tandem Repeat (STR) analysis was performed on PC3 (UT) and PC3 (EMC), confirming these cell lines are all PC3 cells (ATCC). This analysis also confirmed that the used PC3-9 cells are a subclone of the PC3 cell line, as indicated by their STR profile.

### EpCAM expression of patient CTCs

We evaluated the EpCAM expression of CTCs from 13 metastatic prostate cancer patients undergoing Diagnostic LeukApheresis (DLA). CTCs were enriched from DLA derived samples by immunomagnetic depletion of leukocytes with an efficiency ranging from 77.2 to 97.1% (mean 90.3%, SD 7.4%). An overview of the input sample and depletion efficiency of all patients is shown in Supplementary Table S2. After depletion, the samples were stained with CD45-, CD16-, biotin-FITC, Hoechst, Cytokeratin-APC, PSMA-PerCP/Cy5.5 and EpCAM-PE. After washing the samples were resuspended into 1 mL PBS/1%BSA and run on a FACS Aria II (BD Biosciences) using a threshold on Hoechst to only include nucleated events. An example of the flow cytometric measurement is shown in Supplementary Fig. S4. Events were considered as CTC when negative for CD45/CD16/biotin and positive for Hoechst, cytokeratin and PSMA. Although the addition of PSMA positivity in the criteria for CTC identification increases the certainty of their correct identification, some CTCs will be missed by this approach, as not all CTCs are PSMA positive. The EpCAM expression was then quantified on the identified CTCs. To see if the measured intensities could be directly compared to those measured in cell line measurements, antigen expression of PC3 and LNCaP cells was measured after spiking cells into DLA material and compared to that of the same cells measured directly. Results shown in Supplementary Fig. S5 indicate that although there is a difference in the measured number of antigens per cell, this difference is much lower than the differences seen between cell lines as well as between cell lines and patient CTCs.

**Figure 5:**
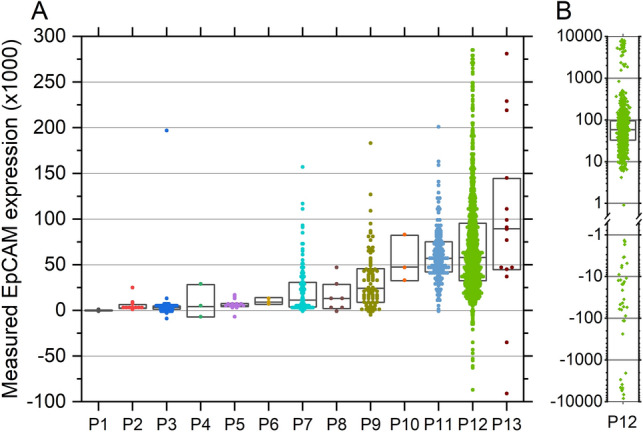
EpCAM expression of CTCs found in leukapheresis products of 13 castration-sensitive prostate cancer patients. (**A**) Expression of CTCs recovered from patients P1-P13 on a linear scale showing a large variation both between patients as well as within a patient. (**B**) Expression of CTCs recovered from patient P12 on a logarithmic scale. Boxes indicate median ± SD. Due to the compensation applied for autofluorescence some cells have attained a negative measured expression.

Figure 5A shows the measured EpCAM expression levels of the individual CTCs found in 13 prostate cancer patients. Per patient a median number of 15 CTCs was measured (mean 148 CTCs, range 2–1457 CTCs). As the EpCAM expression on CTCs of patient 12 extends beyond the scale of Fig. 5A, it is also shown separately on a log scale in Fig. 5B. The measured EpCAM expressions show a large variation, both within and between patients, with the median per patient ranging from 35 to 89,534 (mean 24,993, SD 28,521). In Supplementary Table S2 the mean, median and CV of the measured EpCAM expression of all patients is shown. Here also the relative recovery of CTCs compared to the amount of PSMA positive CTCs found using the CellSearch system is shown.

**Figure 6:**
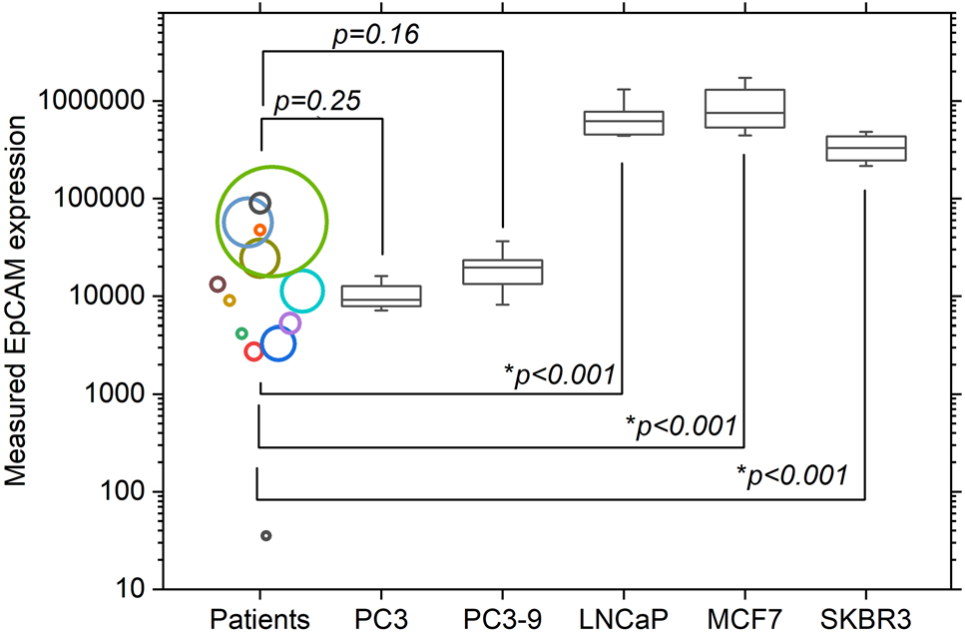
EpCAM expression of CTCs from 13 prostate cancer patients compared to expression measured in five cell lines as cultured at the University of Twente. Circle position indicates median expression per patient and circle size relates to the number of CTCs measured from each patient. Boxes indicate 25–75 percentile with the median as a horizontal line, whiskers indicate 10–90 percentile. * indicates significant difference, *p*-values as determined using the non-parametric Kolmogorov–Smirnov test.

To see how the EpCAM expression of patient CTCs relates to that of often used cancer cell lines, we combined the measured EpCAM expression of all patient CTCs and compared this to the EpCAM expression of the five different cancer cell lines as measured over a 6 to 20-month period, as shown in Fig. 6. Here each circle indicates the measured EpCAM expression in a separate patient, while the circle size is related to the number of CTCs measured. Shown *p*-values were determined using the non-parametric Kolmogorov–Smirnov test. It can be seen that the PC3 and PC3-9 cell lines as cultured at the University of Twente show a similar expression as patient CTCs, while the other three cell lines show much higher EpCAM expressions, making them unfit as a CTC surrogate in the evaluation of EpCAM based enrichment or identification methods.

## Materials and methods

### Cell lines

The prostate carcinoma cell lines PC3 and LNCaP and breast carcinoma cell lines MCF7 and SKBR3 were obtained from ATCC (Manassa, VA, USA). The PC3-9 cell line^24^, a sub-clone of the PC3 cell line, was kindly provided by Immunicon (Huntingdon Valley, PA, USA). PC3, PC3-9 and LNCaP were cultured in RPMI1640 (Lonza), MCF7 and SKBR3 in DMEM (Lonza), all supplemented with 10% FBS (Sigma) and 1% Pen/Strep (Lonza). Cells were cultured at 37° in a humid atmosphere and trypsinized when reaching 70–80% confluence using 0.05% trypsin–EDTA (Gibco, Life Technologies, Waltham, MA, USA).

### Patient samples

In this study, we included 13 patients for the measurement of EpCAM expression on CTCs. All of these patients are newly diagnosed and have not yet received any treatment at the time of sample collection. These patients (age 60–79 years, median 70 years) are all metastatic castration-sensitive prostate cancer patients who have been included into the ongoing PICTURES-study based on the presence of > 2 CTCs in a 7.5 mL sample of blood. In one of the spiking experiments material was used from a patient in the SIBYLLA study, in which breast cancer patients who completed adjuvant endocrine treatment and have no clinical signs of recurrence are included.

Leukapheresis was performed using a Terumo Spectra Optia according to the optimized procedure described by Mout et al.^29^. Samples were collected in accordance with the Declaration of Helsinki as part of studies approved by the medical ethical committee of the Erasmus Medical Center (PICTURES study [MEC20-0422] and SIBYLLA study [MEC20-0384]). Healthy blood samples were collected from anonymized healthy volunteers in a CellSave collection tube (Menarini-Silicon Biosystems) at the University of Twente. In agreement with the Declaration of Helsinki, informed consent was obtained from all volunteers and the used blood collection procedure was approved by the local Medical Research Ethics Committee (METC Twente).

### Expression measurements

Expressions were measured on unfixed cells or when stated on cells fixed with CellSave or 1% formaldehyde. Expressions were determined by labelling the antigen using either an unconjugated or a PE-conjugated antibody for 30 min at 37°. In the case of an unconjugated antibody, cells were washed with PBS/1% BSA and stained with a secondary anti-mouse-PE antibody (12-4010-87, Fisher Scientific) for 30 min at 37°. EpCAM was measured using the anti-EpCAM antibody VU1D9 either unconjugated (kind gift from Immunicon, Huntingdon Valley, PA, USA) or PE-conjugated VU1D9 (SAB4700425-100TST, Sigma). Her2 expression was measured using unconjugated Her2 (kind gift from Immunicon, Huntingdon Valley, PA, USA). PSMA expression was measured using PSMA-PE (342504, BioLegend) and cytokeratin was measured using CK11-PE (5075S, Cell Signaling Technologies). The stained cells were then washed by centrifugation and resuspended in PBS/1% BSA, after which the PE signal intensity of 10,000 cells was measured on a flow cytometer (FACS Aria II, BD Biosciences). The number of PE molecules per cell was calculated using the PE Fluorescent Quantitation Kit (340495, BD Biosciences). Each tube of this kit contains a lyophilized pellet of beads conjugated with four known levels of PE. Using the geometric mean intensity of these four populations we created a calibration curve according to the suppliers’ instructions. Using this calibration curve we then determined the number of PE molecules on the measured cells with the use of the geometric mean value. In experiments containing samples with secondary staining the geometric mean PE intensity of a sample stained using only the secondary antibody was deducted from the measured intensities to correct for non-specific staining. In measurements using directly conjugated antibodies the signal of an unstained sample was used for this correction.

### Cell lines from different institutions

To compare different cultures of the same cell lines as present in different institutions, an aliquot of each cell line was frozen in liquid nitrogen and sent to the University of Twente, while also a new vial of each cell line was ordered at the ATCC. In Supplementary Table S1 an overview of the culture conditions and passage numbers for each cell line and institution are shown. After arrival, all cells were cultured for approximately two weeks in RPMI1640 (LNCaP, PC3) or DMEM (MCF7, SKBR3) (Lonza) supplemented with 10% FBS (Sigma) and 1% Pen/Strep (Lonza), after which they were re-frozen into liquid nitrogen at the University of Twente. For each cell line, all cultures were simultaneously defrosted and cultured for one to two weeks under the same conditions, after which the expression of all antigens was measured in a single experiment.

### Effect on immunomagnetic capture

To illustrate the effect of the different levels of antigen expression on immunomagnetic capture, we spiked a known number of PC3 cells obtained from different institutions into 7.5 mL of healthy donor blood. For this, PC3 cells were stained with Hoechst and brought to a concentration of approximately 50 cells per µL. Three to five one µl droplets were placed on a glass slide and imaged. Using these images, the number of cells in each droplet was counted manually after the experiment. The droplets were rinsed into a 7.5 mL blood sample using 4 mL of CellSearch dilution buffer, after which the glass slide was examined for remaining cells. Subsequent enrichment and staining were performed using the CellSearch system (Menarini-Silicon Biosystems) with the regular CTC Kit. The number of PC3 cells found after scanning and analysis with the CellTracks Analyzer II (Menarini-Silicon Biosystems) was used to calculate recovery.

### EpCAM expression of patient CTCs

To evaluate EpCAM expression on CTCs from cancer patients, we enriched CTCs from DLA material of 13 prostate cancer patients by the depletion of leukocytes. For this, an aliquot of a DLA sample obtained at the Erasmus MC was fixed with CellSave preservative and shipped overnight to the University of Twente. Aliquots of 200 million white blood cells (WBCs) were also processed at the Erasmus MC using the CellSearch system. Here PSMA was added as an additional marker, allowing the comparison of the number of DAPI + CK + PSMA + CD45-CTCs found after depletion of leukocytes to that found using the CellSearch system. For the depletion performed at the University of Twente, a sample aliquot consisting of on average 530 × 10^6^ WBCs (range 230 × 10^6^ to 842 × 10^6^ WBC) was incubated with CD45-Biotin (4 µg/mL, produced and coupled in our institution), CD2-Biotin (555325 BD Pharmingen), CD16-Biotin (555405 BD Pharmingen) and CD19-Biotin (555411 BD Pharmingen) for 15 min. After the addition of 9 mL casein buffer (7 mM sodium phosphate, 41.5 mM sodium chloride, 0.1% sodium azide, 50 mM EDTA, 0.5% BSA, 0.2% mouse serum, 0.5% casein, pH = 7.5) the sample was washed by centrifugation at 800 G for 10 min and resuspended in 6 ml of casein buffer. To this, streptavidin-ferrofluids were added (18 µg/mL, BioMagnetic Solutions) and the sample was incubated for 15 min at room temperature. The separation of bound cells was achieved by placing the sample in a quadrupole magnet for 10 min. Subsequently, the unbound fraction was removed using a glass pasteur pipet connected to a syringe pump (Harvard Apparatus). The pasteur pipet was placed in the centre of the tube and aspiration was performed at a speed of 2 mL/min. Magnetic incubation and aspiration were repeated several times when needed to reach a sufficient depletion level. For the identification of CTCs the depleted sample was stained using a staining cocktail consisting of Hoechst (H3570, Invitrogen) to stain all cells, CK11-APC (1A-108-C100, Exbio), PSMA-PerCP/Cy5.5 (342512, BioLegend), and VU1D9-PE (SAB4700425-100TST, Sigma) for the positive identification of CTCs and CD45-FITC (11-0459-42, eBioscience), CD16-FITC (302,006, BioLegend), and Streptavidin-FITC (S3762-0.1MG, Merck) to stain the remaining white blood cells in the depleted sample. Here the Streptavidin-FITC is used to stain any CD2-, CD16-, CD19- or CD45-biotin coupled to the cells during the depletion procedure, that had not been labelled with a magnetic particle. All staining reagents were combined in a buffer containing 0.05% saponin to permeabilize the cells. Several controls were taken along to allow for a correct compensation and gating (LNCaP and PC3-9 stained with CK11, PSMA, VU1D9 and Hoechst respectively; DLA stained with CD45/CD16/Streptavidin and Hoechst respectively; unstained samples of DLA, LNCaP and PC3-9). The stained cells were then washed and resuspended in PBS/1% BSA and fluorescent intensity was measured on a flow cytometer (FACS Aria II, BD Biosciences). CTCs were identified as CD45/CD16/Strep−, CK/PSMA+ cells. The EpCAM-PE signal was quantified using the PE Fluorescent Quantitation Kit (340495, BD Biosciences), see also Supplementary Fig. S4. As the procedure for depletion, the large background of unwanted cells as well as the higher complexity of the staining mix are expected to influence the measured EpCAM expression, we spiked DLA from prostate (PICTURES study) or breast cancer (SIBYLLA study) patients with approximately 10,000 PC3 or LNCaP cells before performing the depletion and staining procedure, and compared the measured expressions of the spiked cells to that of non-spiked cells measured in the same experiment.

### Statistics

The correlation between EpCAM expression and CellSearch recovery was determined after log-transformation of the EpCAM expression.

In order to obtain a normal distribution, the log-normal distributed antigen expressions were log-transformed and compared using a one way ANOVA. As Levene’s test indicated a homogeneous variance, we performed Bonferroni’s test to determine statistical differences between all groups.

When comparing antigen expression between a single pair, a two sample t-test was performed on log-transformed data.

In cases where paired samples from different cell lines, antigens or preparation methods were grouped, statistical differences were determined using the non-parametrical Wilcoxon Signed Rank Test.

To compare the EpCAM expression of CTCs to that of different cell lines, the non-parametric Kolmogorov–Smirnov test for unpaired samples was used. All statistical and correlation analysis was performed using Origin 2019b (OriginLab Corporation, Northampton, MA, USA) and a significance threshold of 0.05 was used.

### Institutional review board statement

The study was conducted in accordance with the Declaration of Helsinki and approved by the Ethics Committees of the PICTURES (MEC 20-0422) and SIBYLLA (MEC 20-0384) study.

### Informed consent

Informed consent was obtained from all subjects involved in the study.

## Discussion

To effectively develop, compare and test antigen based enrichment assays, the antigen expression of the used CTC surrogate will need to realistically represent real CTCs. Here, the most important characteristic is the expression of the targeted antigen. We have compared the expression of the often targeted EpCAM antigen in CTCs derived from prostate cancer patients with the EpCAM expression of several well-known cell lines using quantitative flow cytometry. Here it is important to realize the measured expression of any antigen using quantitative flow cytometry will depend on a multitude of factors, of which we have only shown a limited number. One of these factors is the ratio between the number of fluorescent molecules bound to a cell and the actual number of antigens. This ratio is determined by the labelling efficiency of the antibody as well as the number of fluorescent molecules per antibody-fluorophore conjugate. The former is dependent on several factors, including the used antibody clone, antibody concentration, incubation time and incubation temperature, but complete labelling is never reached^30^. The latter can be seen in the difference in direct versus secondary antibody staining (Fig. 1A).

Although unfixed cells are the best way of measuring any cell characteristic, in practice patient samples are often processed the next day. In this regard, using a mild fixative such as CellSave has shown to be a good way of preserving the antigen expression, while a stronger fixation (formaldehyde) seems to make the antigens less accessible to the detection antibody. Additionally, stronger fixation causes permeabilization with 0.05% saponin to be ineffective, preventing intracellular staining.

As the EpCAM expressions measured in CTCs are relatively low, the experimental variation between these measurements is also expected to be similar to that observed for PC3-9 (Supplementary Fig. S2).The measured expression range however is relevant, considering that the variation in EpCAM expression seen between patients is still much lower than the EpCAM expression differences seen between patient CTC and some of the tested cell lines.

Additionally, in the independent measurements taken at longer time intervals, the variation seen in the measurement of fixed cells indicate that there is also experimental variation. As however the patient CTCs were measured at 13 different time points during the same period as the cell line expressions were measured, it is expected that the effect of this experimental variation on both sets of measurements is comparable.

Several differences in the expression profiles of the same cell lines cultured at different institutions are found, in some cases despite having a similar passage number and being cultured using the same culture medium. The proliferation of different subtypes of PC3 cells at the Erasmus and University of Twente for instance, shows that using the same culture medium is not the only factor of importance. Likely also other factors such the passage rate, seeding density and culture flask type are of influence.

Some of the CTCs show a measured EpCAM expression below zero. This is due to the correction applied for the background signal present in the flow cytometer. As we apply a fixed correction for each cell based on the geometric mean intensity of the negative control sample, for approximately half of the cells this correction will be larger than their actual background signal, causing in some cases the calculated amount of PE-molecules per cell to become negative. Similarly, approximately half of the cells are corrected less than their actual background signal. To keep the variation evenly distributed we have chosen not to set these negative values to zero.

As can be seen in Fig. 6, out of the cell lines tested, the EpCAM expression of PC3 and PC3-9 cells is most similar to that of CTCs derived from prostate cancer patients. The only other study that to our knowledge has attempted to quantitatively measure the EpCAM expression on CTCs is that of Rao et al.^24^. Their results show a higher measured EpCAM expression (49,708 antigens per cell) compared to our findings. These expressions however cannot be directly compared as Rao et al. did not only use CTCs from prostate cancer patients, while they also used a minimal EpCAM expression threshold in their CTC selection, effectively excluding any EpCAM negative CTCs. Interestingly however, they also determined the EpCAM expression of the PC3-9 cell line to be suitable as a CTC representative, which is in part due to their culture of PC3-9 cells having a higher EpCAM expression compared to ours.

When relating the EpCAM expression of CTCs to the CellSearch recovery using the linear regression depicted in Fig. 4, it becomes apparent that based solely on their EpCAM expression, the expected recovery of CTCs for some patients is in the range of 1–5%. In previous studies, we have detected additional CTCs in the waste of the CellSearch system^13^, but not to this extent. We expect the reason for this to be that CellSearch recovery is not only dependent on the EpCAM expression, but that other characteristics such as size are also important in the immunomagnetic capture of CTCs. To be captured, the relatively large PC3 cells will therefor likely need more EpCAM expression than an on average smaller prostate cancer CTC does.

In this regard, our expectation was also to see a correlation between the EpCAM expression and the relative recovery of CTCs using a depletion approach compared to CellSearch enrichment. In the 13 measured patients, we have however not observed such a relation (see data in Supplementary Table S2). This is likely in part due to the uncertainty of the exact level of EpCAM expression in these patients, as well as a variation in the CTC loss during the depletion and staining procedures. Another factor that might cause more CTCs to be measured in some patients using the flow cytometer is its higher sensitivity, causing cells with equal PSMA expression to be scored as PSMA negative in CellSearch, while they are deemed positive in the more sensitive flow cytometry measurement.

Although clearly not the only factor of importance, when choosing a cell line for optimization one should aim to use one or more cell lines in the range of expression that is measured in real CTCs. As can be seen in Fig. 3, this does not mean that PC3 cells purchased from the ATCC are suitable as a low expressing CTC equivalent, as their expression is at the top of what is measured in real CTCs. This difference also shows the need for the regular quantification of antigen expression if comparisons using cell lines are to be made.

## Conclusions

In this work, we have shown that comparing antigen-based cell enrichment methods at different institutions using the same cell lines can result in large differences in capture efficiency, as cell lines cultured at different institutions show a large variation in antigen expression. Ideally, a cell line with an expression comparable to real CTCs is used. For EpCAM, we show an expression on CTCs that differs between castration sensitive prostate cancer patients, but is much lower than many of the cell lines often used to evaluate CTC enrichment technologies.

## Supplementary Information

Supplementary Information.

## Data Availability

The datasets generated during the current study are available from the corresponding author on reasonable request. All flow cytometry data used in this manuscript is available in standard .fcs file format. Images used for counting of spiked cells as well as the CellSearch scans used to evaluate recovery are available in the original .tiff format.
