# Supplementary material for: Measuring antigen expression of cancer cell lines and circulating tumour cells
Source: Sci Rep. 2023 Apr 13;13:6051. doi: 10.1038/s41598-023-33179-y (PMC10101999; doi:10.1038/s41598-023-33179-y)
Supplement: Supplementary file 1 — Supplementary Information. [file 41598_2023_33179_MOESM1_ESM.docx]

Supplementary Information

*Measuring antigen expression of cancer cell lines and circulating tumour cells*

Anouk Mentink ^1^, Khrystany T. Isebia ^2^, Jaco Kraan ^2^, Leon W.M.M. Terstappen ^1^ and Michiel Stevens ^1,^*

^1^ Medical Cell Biophysics Group, Techmed Center, Faculty of Science and Technology, University of Twente, PO Box 217, 7500AE Enschede, The Netherlands

^2^ Department of Medical Oncology, Erasmus MC Cancer Institute, Erasmus University Medical Center, Rotterdam, the Netherlands; Cancer Genomics Netherlands, Erasmus MC Cancer Institute, Erasmus University Medical Center, Rotterdam, the Netherlands.

***** Correspondence: Michiel Stevens, email: [m.stevens@utwente.nl](mailto:m.stevens@utwente.nl), tel: +31534894101

**
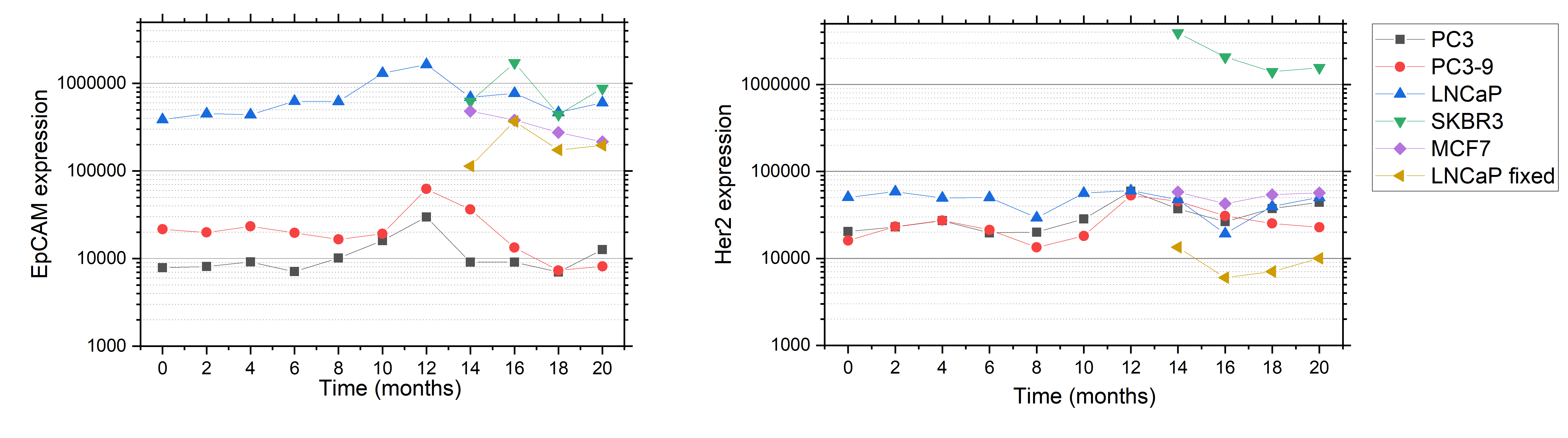
**

**Figure S1**. EpCAM and Her2 expression measurements were performed every 2 months during a 6 (SKBR3, MCF7, and LNCaP fixed) or 20 (PC3, PC3-9, and LNCaP) month period. Data is shown in relation to time, showing a similar pattern in multiple cell lines, in line with the notion that the variation is mostly caused by experimental variation.

**
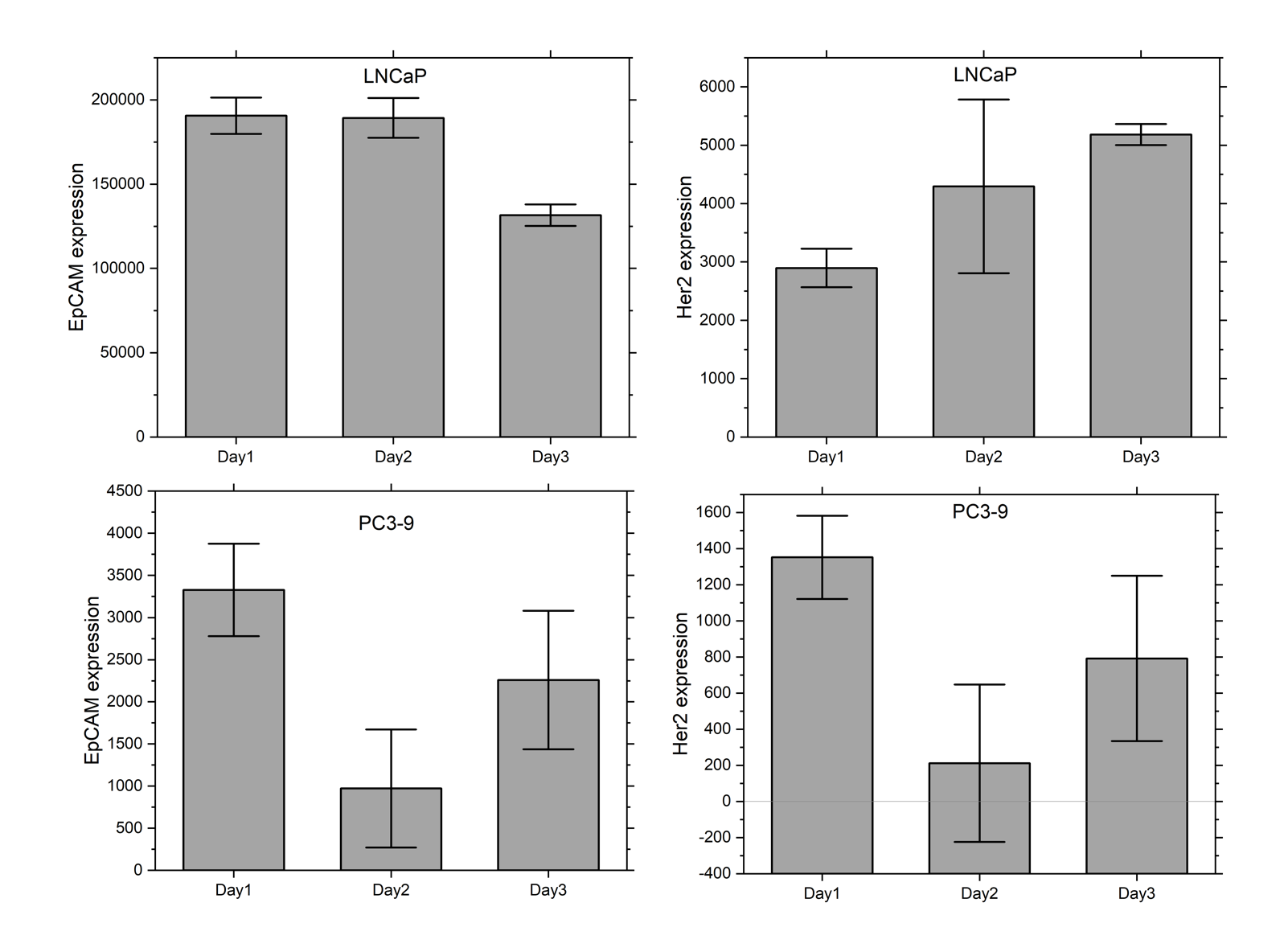
Figure S2**. EpCAM and Her2 expression measurements were performed on the same day. Each bar indicates the mean of three independent samples of LNCaP and PC3-9 cells as measured on the same day (day 1). The experiment was repeated twice within a week (day 2 and day 3). Error bars indicate the standard deviation.


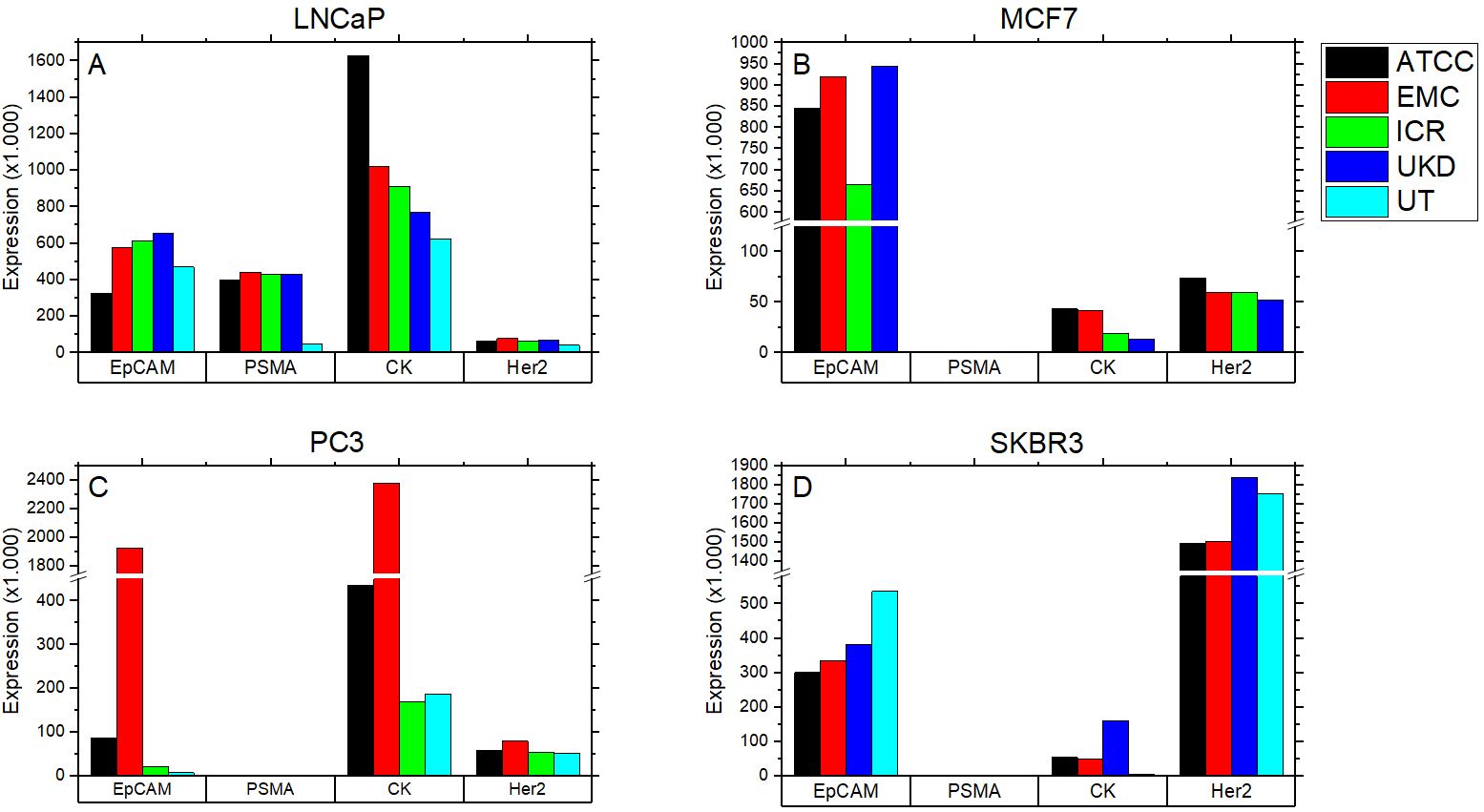


Figure S3. The expression of EpCAM, Her2, PSMA and Cytokeratin (CK) in LNCaP, MCF7, PC3 and SKBR3 from the ATCC, Erasmus MC (EMC), London Institute for Cancer Research (ICR), Universitätsklinikum Düsseldorf (UKD) and University of Twente (UT).


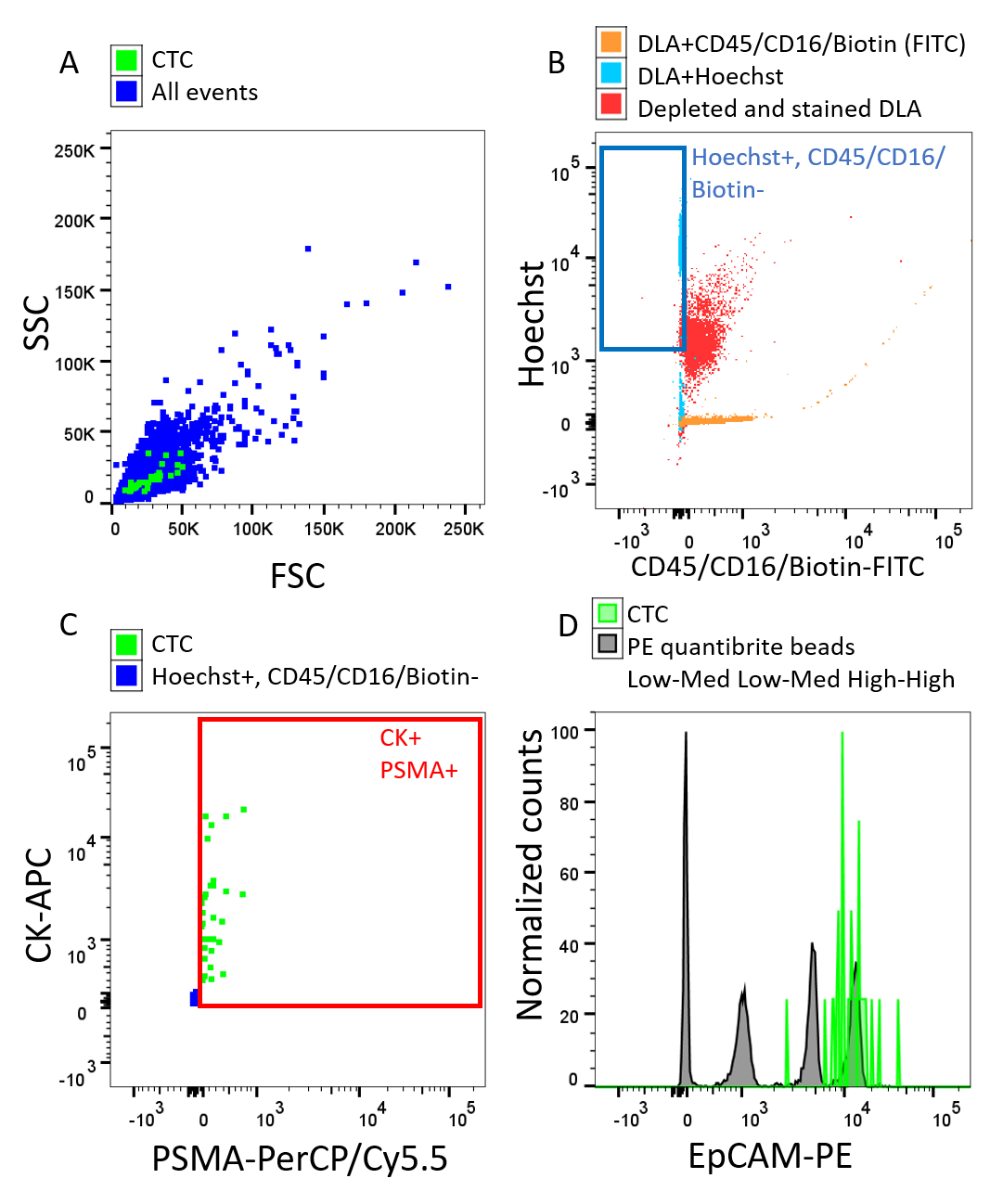


Figure S4. CTC selection using flow cytometry. FSC/SSC plot where CTCs (green) are shown (A). CTCs were selected based on control samples stained with Hoechst and CD45/CD16/Biotin-FITC respectively (B). In the Hoechst+, CD45/CD16/Biotin- population, CTCs were gated based on cytokeratin and PSMA staining (C). EpCAM expression of CTCs was measured and calculated using PE Quantibrite beads (D).


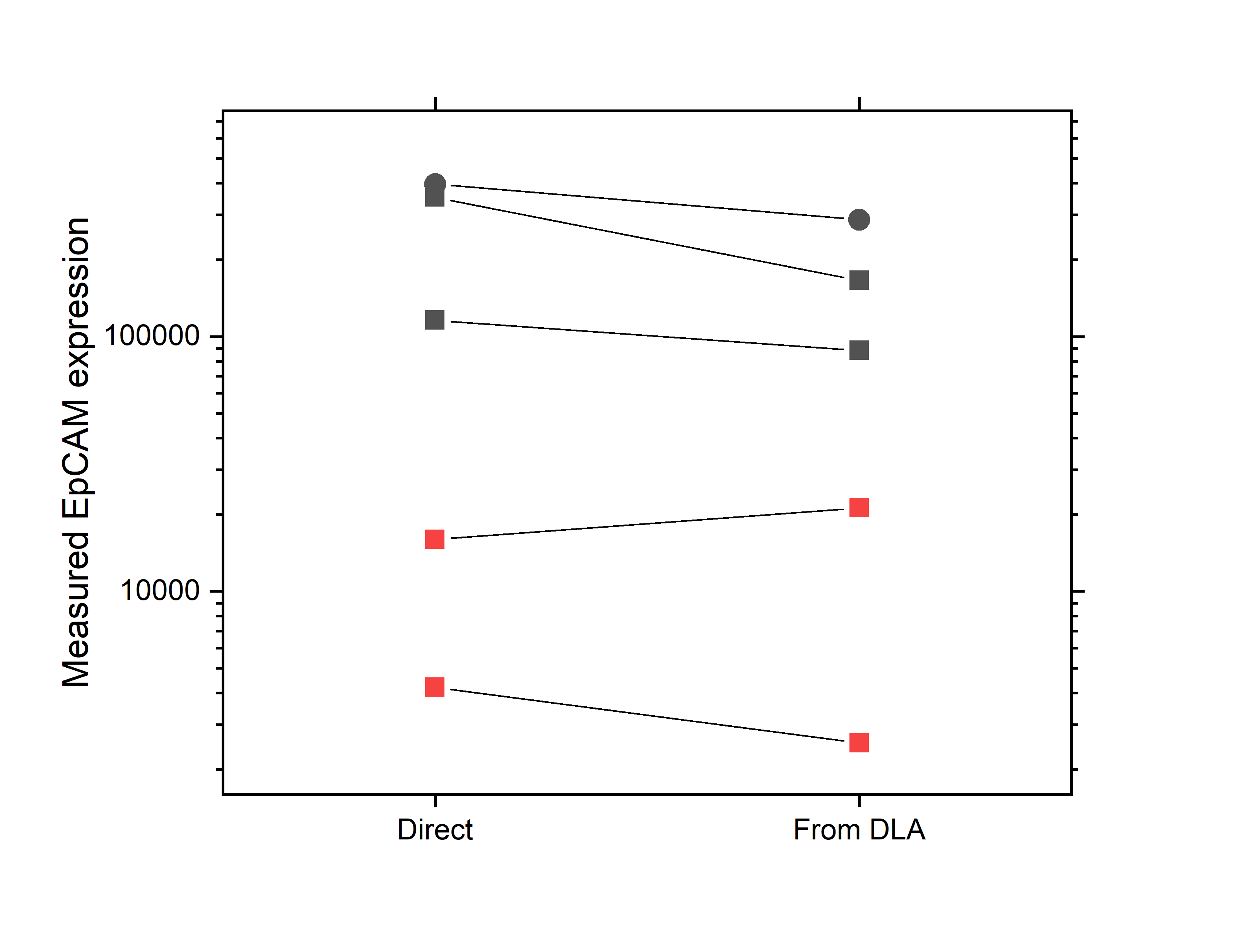


**Figure S5**. Measured EpCAM expression of LNCaP and PC3 cells measured directly or measured after spiking into DLA. Red markers indicate PC3 cells (N=2), black markers indicate LNCaP cells (N=3). Squares indicate 1% formaldehyde fixed cells (N=4), while circles indicate CellSave fixed cells (N=1).

| Cell line | Institution | Culture medium | Supplements (All: antibiotics) | Passage during measurement |
| --- | --- | --- | --- | --- |
| LNCaP | ATCC | RPMI1640 | 10% FBS | 25 |
|  | EMC | RPMI1640 | 10% FBS | 16 |
|  | ICR | RPMI1640 | 1% FBS, 1% Glutamax | 16 |
|  | UKD | RPMI1640 | 20% FBS | >25 |
|  | UT | RPMI1640 | 10% FBS | 87 |
| MCF7 | ATCC | EMEM | 10% FBS, 0,01 mg/ml insulin | 154 |
|  | EMC | RPMI1640 | 10% FBS | 159 |
|  | ICR | DMEM | 10% FBS | >4 |
|  | UKD | RPMI1640 | 10% FBS | >5 |
| PC3 | ATCC | F12-K | 10% FBS | 28 |
|  | EMC | RPMI1640 | 10% FBS | 28 |
|  | ICR | RPMI1640 | 1% FBS, 1% Glutamax | 8 |
|  | UT | RPMI1640 | 10% FBS | 30 |
| SKBR3 | ATCC | McCoy’s 5A | 10% FBS | 34 |
|  | EMC | RPMI1640 | 10% FBS | 53 |
|  | UKD | McCoy’s 5A | 10% FBS | >8 |
|  | UT | DMEM | 10% FBS | 195 |

Table S1. For each cell line the culture medium, supplements and passage during measurement are shown. When the passage number is unknown, we show the minimal passage the cells have (e.g. >4 means passage 4 or higher).

| Patient | Median  EpCAM  expression | Mean  EpCAM expression | St dev  EpCAM expression | CTCs  found | PSMA+ CTCs in CellSearch | Relative % of CTC  recovery | Depletion input (10^6 WBC) | Depletion efficiency % | Cells  measured |
| --- | --- | --- | --- | --- | --- | --- | --- | --- | --- |
| P1 | 35 | 35 | 647 | 2 | 27 | 85% | 381 | 96% | 731096 |
| P2 | 2714 | 5439 | 6684 | 11 | 10 | 470% | 320 | 97% | 1360837 |
| P3 | 3279 | 6159 | 25392 | 59 | 8 | 2907% | 500 | 96% | 2029451 |
| P4 | 4176 | 8377 | 18063 | 3 | 5 | 175% | 519 | 95% | 3152160 |
| P5 | 5275 | 6431 | 5620 | 15 | 45 | 811% | 495 | 77% | 1871481 |
| P6 | 8995 | 9823 | 3764 | 3 | 254 | 16% | 842 | 90% | 1524868 |
| P7 | 11261 | 23441 | 28299 | 106 | 1973 | 74% | 606 | 87% | 1932223 |
| P8 | 13173 | 15161 | 17319 | 7 | 13 | 154% | 718 | 95% | 3542436 |
| P9 | 24351 | 34126 | 32648 | 82 | 289 | 470% | 760 | 80% | 2413387 |
| P10 | 47341 | 54062 | 25556 | 3 | 0 | - | 509 | 93% | 930840 |
| P11 | 56935 | 61018 | 31018 | 158 | 385 | 32% | 675 | 97% | 8566310 |
| P12 | 57840 | 142980 | 885522 | 1457 | 518 | 813% | 230 | 93% | 4909001 |
| P13 | 89534 | 92798 | 97280 | 15 | 20 | 1374% | 325 | 78% | 2420725 |

Table S2. For each patient the median, mean and standard deviation of the measured EpCAM expression, number of CTCs measured in flowcytometry, number of PSMA+ CTCs found in a standard CellSearch run, relative CTC recovery compared to CellSearch based on WBC input and sample input, depletion efficiency and number of cells measured in flowcytometry are shown.
